# Challenges and directions: an analysis of Genetic Analysis Workshop 17 data by collapsing rare variants within family data

**DOI:** 10.1186/1753-6561-5-S9-S30

**Published:** 2011-11-29

**Authors:** Peng Lin, Michael Hamm, Sarah Hartz, Zhehao Zhang, John P Rice

**Affiliations:** 1 Department of Psychiatry, Washington University, 660 S. Euclid Ave., Campus Box 8134, St. Louis, MO 63110, USA

## Abstract

Recent studies suggest that the traditional case-control study design does not have sufficient power to discover rare risk variants. Two different methods—collapsing and family data—are suggested as alternatives for discovering these rare variants. Compared with common variants, rare variants have unique characteristics. In this paper, we assess the distribution of rare variants in family data. We notice that a large number of rare variants exist only in one or two families and that the association result is largely shaped by those families. Therefore we explore the possibility of integrating both the collapsing method and the family data method. This combinational approach offers a potential power boost for certain causal genes, including *VEGFA*, *VEGFC*, *SIRT1*, *SREBF1*, *PIK3R3*, *VLDLR*, *PLAT*, and *FLT4*, and thus deserves further investigation.

## Background

Genome-wide association studies have accelerated the discovery of genetic variants that cause disease. Thus far, nearly 600 genome-wide association studies have examined about 150 distinct diseases or traits, and more than 800 single-nucleotide polymorphism (SNPs) associated with these diseases or traits have been identified [1]. Recent studies have suggested that rare variants contribute to common diseases, but the case-control study design does not have sufficient power to discover rare causal variants.

Two common approaches are used to increase the power to detect rare variants. One method is to collapse rare variants on the basis of predetermined criteria. By grouping risk variants together, the frequency of rare risk variants can be increased in the data set. Extensive research on collapsing has been done for population-based data [2]. Another approach is to examine family data. The potential advantage of family data is that a particular rare variant found in an affected individual is more common in that individual’s family than in subjects randomly sampled in the population.

Genetic Analysis Workshop 17 (GAW17) is a collaborative effort among researchers to improve our current understanding of genetic architecture. It provides simulated data based on real exon sequence data and thus offers a unique and relatively realistic opportunity to evaluate statistical genetic methods that are relevant to current analytical problems. For this workshop, we designed a study to (1) test both the collapsing method and the family design in data sets generated with the same biological model and (2) assess the power of combining these two approaches (collapsing rare variants within family data). This study will help guide researchers to design and analyze future studies for the detection of rare genetic variants.

## Methods

### Family-based association testing

To test genetic associations in family data, investigators need to address the correlation among family members. Several methods are available [3-5]. We accounted for correlated genotypes by using the modified quasi-likelihood score test (*M*_QLS_) developed by Thornton and McPeek [4]. This method is implemented in the computer program M_QLS_.

*M*_QLS_ is an improvement on the previous quasi-likelihood score test, *W*_QLS_, developed by Bourgain et al. [6]. It accounts for the correlations among related individuals by using a defined kinship matrix and assigns optimal weights depending on the pedigree information, thus providing an efficient estimator of allele frequency under the null hypotheses. Interested researchers should refer to Thornton and McPeek’s paper for more details [4].

### Collapsing rare variants within family-based association testing

A causal gene can be shared by more than one or two families, although this gene can have different rare risk variants in those families. Traditional family-based association tests fail to combine signals from different rare variants. To address this issue, we proposed to collapse these rare variants. Many collapsing methods are available. Some methods simply account for the presence or absence of rare variants, whereas others assign an adjustable weight to different types of rare variants, based on biological function or minor allele frequency, and then calculate a final score for each gene [2]. Currently, there is no conclusive evidence to argue for or against a particular collapsing method. To generate data that can be analyzed by *M*_QLS_, we created a gene indicator that collapses rare variants within the same gene. Similar to SNPs, the gene indicator is a dichotomous variable that indicates presence or absence of any rare variant within the region of interest, so it can be processed by the M_QLS_ program. A gene indicator variable *G* for the *n*th subject is defined as:(1)

Although genotype *BB* can be defined when both alleles of a particular SNP are the rare alleles, the likelihood of this situation is small, because we are dealing with rare variants.

We have developed a SAS macro to implement our method with the M_QLS_ program. The SAS macro is available to interested investigators.

### Power analysis

A subset of genes that had sequence data available in the 1000 Genomes Project was included in this GAW17 project. GAW17 simulated the phenotype based on a predefined simulation model and generated 200 different phenotype files under the same model. Thus the 200 replicate phenotype files provide a unique opportunity to estimate power. We tested associations under different conditions and calculated the power of different approaches. Power is defined as the proportion of times that a particular test reaches the significance threshold.

## Results

### Distribution of rare variants within family data

The GAW17 data set has 697 subjects (209 case subjects and 488 control subjects) from 8 families. A total of 24,487 SNPs were simulated for 3,205 genes. Fully informative identical-by-descent (IBD) scores were also provided for each gene.

**Figure 1:**
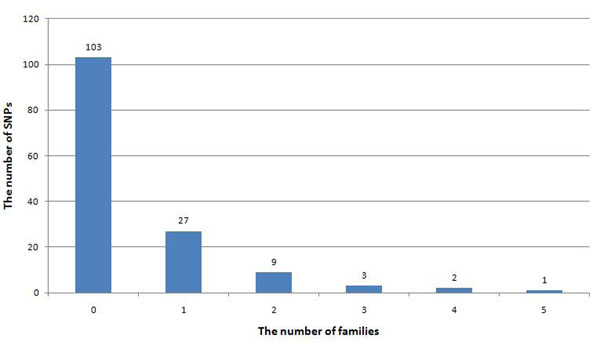
**Distribution of rare causal SNPs within families.** In the GAW17 data set, 145 of 162 casual SNPs are rare variants. Of these 145 rare variants, 103 do not exist in the family data. Eighty-five percent of the existing rare variants exist in only one or two families. The number above each bar indicates the exact number of rare SNPs in this category. It partly explains why many rare variants cannot be discovered using family data.

We defined a SNP as rare if its minor allele frequency (MAF) in the population was less than 0.01. By this definition, in the GAW17 data there are 18,131 rare SNPs, 56.4% of which do not exist in the family data. According to the simulation model, 162 SNPs underlie the disease status. Among them, 145 are rare SNPs. Unfortunately, more than 70% of these rare SNPs do not exist in the family data. In addition, a large proportion (85%) of the remaining SNPs exist in only one or two families (Figure 1).

Moreover, many existing rare variants are not passed on in the family. Analysis of the family data shows that 30 of the 42 rare variants that exist in founders are not passed on to offspring. In fact, only 10 of the 42 rare SNPs (7% of all the causative rare SNPs) have an allele frequency (frequency in family data) greater than 0.01.

### Family-based association test

**Figure 2:**
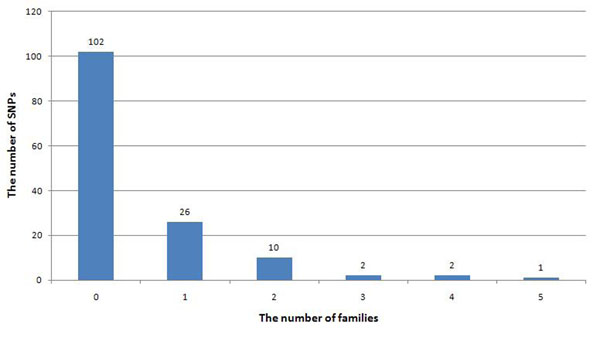
**Distribution of association signals withinfamilies**. Each category indicates the number of families that report an association signal for each SNP. The number above each bar indicates the total number of rare causal SNPs in this category. The distribution of association signals matches well to the distribution of rare SNPs within families. It shows that when all families are analyzed together, the final result is largely shaped by only a few families.

Because 85% of the 36 rare SNPs found in families exist in only one or two families, it is expected that only one or two families can contribute to the final association result. Among the 145 rare SNPs that underlie the disease status, most signals exist in only one or two families. The distribution of signals is shown in Figure 2, and it matches the distribution of rare SNPs within families well.

In addition, combining families that have a particular risk allele with families that do not have the particular risk allele unintentionally diminishes the power. We compared the association result from all families and the association result from each family. Seventy-seven percent of rare causal SNPs have more significant *P*-values from one family than from all data analyzed together.

### Collapsing rare variants within family-based association test

As we have shown, for a particular rare risk variant, only one or two families contribute to the signal, but one gene may have multiple risk variants, each of which may be possessed by different families. Cystic fibrosis transmembrane conductance regulator (*CFTR*) is a good example. Since *CFTR* was identified, more than 1,000 mutations have been found for cystic fibrosis [7]. And similar to *CFTR*, a causal gene may have multiple mutations, and different families may have different risk mutations within the same gene. Because these different mutations can be designated by a risk gene indicator, we believe that collapsing those different mutations to a gene indicator may provide an additional boost on power.

We tested collapsing within family data using the method described in the Methods section. One particular question we want to address here is whether there is any benefit to collapsing within families compared to collapsing in population-based data, which has been extensively researched.

We set our significance level to a loose level of *P* < 0.05 for power calculation and repeated our analysis in the 200 phenotype data sets. We collapsed all rare SNPs (MAF < 0.01) within genes. The SNP for *GCKR* has a MAF greater than 0.01 and thus was excluded from analysis. Among 35 available genes, 17 reached the significance threshold. The power for these genes is shown in Table 1. For comparison, we did similar analyses in the population data with two dummy variables to adjust for ancestry. From the table, we notice that family-based collapsing is more useful for certain genes.

**Table 1:** Comparison of collapsing within family data and collapsing within population-based data

Chromosome	Gene	Number of synonymous SNPs	Number of nonsynonymous SNPs	Total number of SNPs	Number of risk SNPs	Family		Population	
						
						Power (All) (%)^a^	Power (nonsynonymous) (%)^b^	Power (All) (%)^a^	Power (nonsynonymous) (%)^b^
6	*VEGFA*	2	2	4	1	100	100	13	10
4	*VEGFC*	0	1	1	1	100	100	0	0
10	*SIRT1*	9	14	23	9	19	47	7	8
17	*SREBF1*	5	16	21	10	19	36	17	18
1	*PIK3R3*	3	2	5	1	11	1	2	2
9	*VLDLR*	8	15	23	8	10	4	10	9
8	*PLAT*	14	11	25	8	8	34	6	7
5	*FLT4*	3	5	8	2	6	13	15	15
4	*KDR*	5	9	14	8	0	0	45	35
18	*PIK3C3*	5	1	6	1	0	0	38	0
8	*PTK2B*	6	3	9	3	0	0	31	4
14	*SOS2*	1	6	7	2	0	0	22	25
13	*FLT1*	8	17	25	8	2	1	21	17
3	*BCHE*	3	25	26	13	0	1	19	19
11	*PDGFD*	0	6	6	4	6	6	19	19
14	*HIF1A*	2	5	7	3	0	0	17	17
8	*PTK2*	4	5	9	2	0	0	14	6
1	*PIK3C2B*	22	38	60	23	2	0	14	16
1	*SHC1*	3	3	6	1	0	0	13	5
6	*VNN1*	4	2	6	1	1	2	7	13

Power is calculated based on the threshold *P* < 0.05. Because of limited space, only those genes whose power is greater than 10% are shown.

^a^ All rare SNPs were collapsed.

^b^ Only nonsynonymous rare SNPs were collapsed.

Among those genes for which the family-based collapsing has power, we set our significance threshold to the stringent level of 0.05/3,205 = 1.56 × 10^−5^. The power for *VEGFC* and *VEGFA* is 99% and 94.5%, respectively. Population-based collapsing, however, has no power to detect these two genes. Among the 200 phenotypes, the population-based collapsing reported a median *P*-value of 0.98 for *VEGFC* and 0.54 for *VEGFA*.

Another issue we want to address is whether there is any gain in power for collapsing within families compared to the family approach without collapsing. We tested each SNP using *M*_QLS_ within family data. The result is shown in Table 2. The comparison shows that collapsing may be useful for some variants and may be detrimental for some other variants. In fact, collapsing a causal variant with a noncausal variant will diminish power. We found that *SIRT1* and *VLDLR* have a power drop, but for some other genes, such as *SREBF1*, *PIK3R3*, *PLAT*, and *FLT4*, there are considerable power gains. Further analysis shows that among those genes that have power gains by the family-based collapsing, many families that possess different risk variants have contributed to the signal.

**Table 2:** Comparison of family data with collapsing and family data without collapsing

Chromosome	Gene	Number of synonymous SNPs	Number of nonsynonymous SNPs	Total number of SNPs	Number of risk SNPs	Collapsing		Noncollapsing, power (%)
							
						Power (All) (%)^a^	Power (nonsynonymous) (%)^b^	
6	*VEGFA*	2	2	4	1	100.0	100.0	100.0
4	*VEGFC*	0	1	1	1	100.0	100.0	100.0
10	*SIRT1*	9	14	23	9	19.0	47	99
9	*VLDLR*	8	15	23	8	10.0	3.50	58.50
17	*SREBF1*	5	16	21	10	18.5	36	22.50
1	*PIK3R3*	3	2	5	1	11.0	1	0
8	*PLAT*	14	11	25	8	8.0	34	0
5	*FLT4*	3	5	8	2	6.0	12.50	0

Power is calculated based on the threshold *P* < 0.05. Because of limited space, only those genes whose power is greater than 10% are shown.

^a^ All rare SNPs were collapsed.

^b^ Only nonsynonymous rare SNPs were collapsed.

## Discussion

Recent advances in genome-wide association studies have identified hundreds of common SNPs that are associated with different diseases, but collectively they can explain only a small fraction of variation. Many investigators believe that the missing heritability may be partly explained by the rare variants, which are difficult to discover in the common case-control study design. One reason that the existing study design does not have sufficient power is simply because these rare variants are rare. In general, for any statistical test, a certain number of subjects who possess this particular rare variant are required in order to obtain enough power. From this perspective, the family design and the collapsing approach, both of which are potential methods for discovering rare variants, aim to increase the presence or the frequency of the risk variant or haplotype in the data set. However, some challenges are associated with these two methods.

It is generally thought that because a rare mutation can be transmitted to offspring, family data may have more copies of rare mutations than can be found in population-based data. However, a large number of rare mutations that are possessed by founders are not passed on in the family data. Among 145 rare SNPs, only 10 have an allele frequency (frequency in family data) greater than 0.01. This may partly explain the general conclusion reached in the GAW17 meeting that family data are not particularly helpful for discovering rare risk variants.

In addition, collapsing should be used with caution. The assumption behind collapsing is that risk alleles tend to be rare. This assumption may be supported by evolution theory. If one new variant is generated by mutation and is beneficial, then this new variant will be favored by selection and therefore its frequency will increase over time. Similarly, malicious alleles are selected against, and therefore their frequency will decrease over time. Moreover, if a nonsynonymous mutation occurs at a conservative gene coding region, it is likely that the mutation will be malicious, because that is why the sequence is otherwise conservative. However, some neutral rare variants can exist in the population as a result of random mutation. Grouping a risk variant with a neutral variant may decrease the power, as we have shown in Table 1.

In GAW17, all risk variants are nonsynonymous SNPs. In Table 1, the power is lower when collapsing all rare variants than when collapsing only nonsynonymous SNPs. It is tempting to argue that we should collapse only nonsynonymous SNPs. In reality, however, synonymous SNPs may play a significant role in biological function, for example, alternative splice site, transcription factor binding site, or even chromatin structure protein binding site. Meanwhile, nonsynonymous SNPs may have no function at all. At the protein level, an amino acid change, which is usually the result of nonsynonymous SNPs, often fails to change the secondary structure and tertiary structure of a protein and therefore may have no impact on protein function. Although it is generally difficult to predict whether a synonymous SNP or a nonsynonymous SNP is biologically functional or not, we believe that the use of prediction algorithms for function will be helpful. Several function prediction algorithms are available, for example, SIFT and PolyPhen-2 [8,9]. Unfortunately, all causal variants in the GAW17 simulation data were chosen based on PolyPhen and SIFT predictions of the likelihood that the variant would be deleterious. Thus the application of the function prediction algorithm to the GAW17 simulation data, which were generated using the same function prediction algorithm, may not be illuminating.

One purpose of this study is to cast new light on future study designs. We noticed that in family data, the association signals exist in only one or two families. We also noticed that combining these families with families that do not possess these risk variants unintentionally diminishes power. Therefore we argue that, given a limited sample size, a large pedigree may be more useful for discovering rare risk variants. Although many rare variants cannot be discovered, a large pedigree is still useful because at least some causal rare variants are more likely to be found.

In conjunction with association testing, linkage can identify regions of interest. Therefore regional sequencing can be done instead of whole genome sequencing. In addition, the selection of the most informative families or family members may further reduce the total genotyping cost. In addition, the use of extremes of a phenotypic distribution may provide tremendous information and reduce the required sample size [10].

In this study, we tested collapsing within family data, which combines the two widely proposed methods: the family design and the collapsing approach. The new combinational method provides considerable power gain for some genes. Although we noticed that the power gain is obtained at the cost of power for some other genes, this is still useful, especially if the alternative is that nothing can be found. As we have shown in this paper, this method can be useful for discovering novel variants associated with disease, and thus it merits further study.

## Conclusions

Family data are believed to be one way to increase the presence of rare variants in the data set. But a large number of rare risk variants cannot be sampled in the family data. Even for existing rare risk variants, a large proportion of them are not passed on in the family. Many existing rare risk variants are seen in only one or two families, and the result from association is largely shaped by those families. To aggregate signals from different rare variants in different families, we integrated the collapsing method within the family data method. To our knowledge, this is the first attempt in the literature to do collapsing within family data. This combinational approach offers a promising power boost for certain causal genes and thus deserves further investigation.

## Competing interests

The authors declare that there are no competing interests.

## Authors’ contributions

PL designed the method, carried out the analysis and drafted the manuscript. MH carried out the analysis and drafted the manuscript. SH participated in the study design and drafted the manuscript. ZZ participated in performing the statistical analysis. JPR conceived of the study and participated in its design and coordination and helped to draft the manuscript. All authors read and approved the final manuscript.
